# A novel immunoglobulin G monolayer silver bio-nanocomposite

**DOI:** 10.1186/s13065-015-0126-z

**Published:** 2015-10-06

**Authors:** Cristian T. Matea, Teodora Mocan, Florin Zaharie, Cornel Iancu, Lucian Mocan

**Affiliations:** 3rd Surgery Clinic, Department of Nanomedicine, “Iuliu Hatieganu” University of Medicine and Pharmacy, Croitorilor 19-21, 400162 Cluj-Napoca, Romania; Department of Physiology, “Iuliu Hatieganu” University of Medicine and Pharmacy, Clinicilor 5-7, Cluj-Napoca, Romania

**Keywords:** Silver nanoparticle functionalization, Immunoglobulin, Calcium folinate

## Abstract

**Background:**

Nanoparticles have a large number of surface atoms, which translates into a significant increase in the surface energy. Once introduced in a biological environment they tend to interact with proteins and form a protein corona shell. The aim of this study was to develop a novel, silver based, bio-nanocomposite for biological applications. Immunoglobulin G (IgG) molecule was chosen for the passivation of the silver nanoparticles (AgNPs) in order to avoid macrophage recognition of the synthesized structures.

**Results:**

Monodisperse IgG-folinate functionalized silver nanoparticles were obtained, with sizes around 39 nm. UV–Vis and UATR-FT-IR spectroscopies were employed to confirm the successful functionalization of the silver nanoparticles. Atomic force microscopy and dynamic light scattering measurements gave information about the size and shape of the nanoparticles prior and after the passivation with IgG.

**Conclusions:**

Immunoglobulin G formed a monolayer around the nanoparticles with the binding site seemingly in the Fc domain, leaving the two Fab regions available for antigen binding. To our knowledge, this is the first report of an IgG-folinate functionalized AgNP bionanostructure developed for biological applications.

**Graphical abstract:**
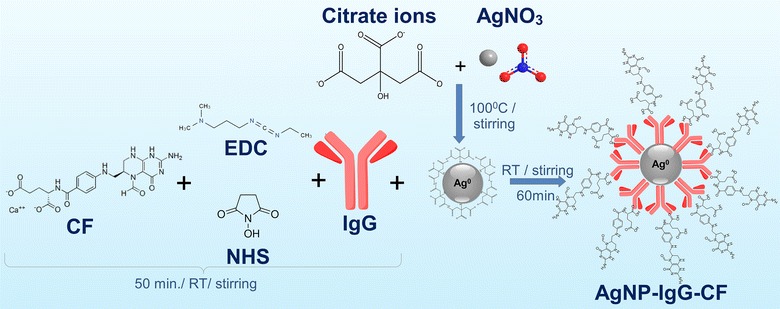
Graphical illustration for IgG-folinate silver nanoparticles functionalization steps

## Background

In the past few decades, metal nanoparticles have been developed for a variety of applications, such as biosensors, anti-bacterial agents, drug-delivery vehicles, contrast agents and so on. Silver nanoparticles (AgNPs) have attracted a lot of attention due to their ease of synthesis, chemical stability, good conductivity and antimicrobial properties [1–3].

A wide variety of methods for silver nanoparticle synthesis is available in literature, these include: chemical reduction, laser ablation, thermal decomposition and sonochemical synthesis [4–8]. A vastly utilized method for silver nanoparticle synthesis is the reduction of silver ions in a aqueous solution in the presence of a capping agent [9], such as citrate molecules which impart negative surface charges that prevent nanoparticle aggregation through repulsion forces [10].

Silver nanoparticles present a surface plasmon resonance band (SPR) derived from the collective oscillation of valence electrons under stimulation from incident light, this phenomenon is observed when the frequency of light photons matches the natural frequency of surface electrons oscillating against the restoring force [11]. The SPR band is sensitive to the nanoparticle size and shape and also to the properties of the surrounding medium [12].

Nanoparticles have a large number of surface atoms, which translates into a significant increase in the surface energy. A tendency to reduce their large surface energy by interacting with the surrounding components that contain donating or accepting sites has been observed [13]. Literature data shows that nanoparticles introduced in a biological environment tend to interact with proteins and form a protein corona shell [14]. The nature of the protein corona influences the extent and mechanism of nanoparticle cellular internalization [15, 16]. AgNPs that are stable enough to significantly restrict bacterial growth are challenging to prepare [17]. Thus, the passivation of nanoparticles, a technique that involves surface modification or coating with naturally-occurring molecules (such as serum abundant proteins or fatty acids) has to be taken into account when designing nanoparticles for life sciences applications [18, 19].

It has been showed that when the protein concentration is sufficient to cover the available silver nanoparticle surface, the protein molecules at the metal–water interface retain their native structure [20].

Silver has a well-documented toxic effect on lower organisms while a biological role in the human body has not yet been demonstrated [21]. Aueviriyavit et al. showed that AgNPs can cause acute cellular damage in Caco-2 (HTB-37) cells but only after treatment with relatively high amounts nanoparticles [22]. Several other studies show that AgNPs can cause apoptosis, DNA damage or cause oxidative stress, but interestingly toxicity levels reported vary greatly [17]. Thus, the AgNPs low toxicity in humans makes them excellent candidates for future silver based drugs in the fight against antibiotic-resistant bacteria [21]. Strong antibacterial activity of AgNPs was demonstrated at very low total concentrations of silver (<7 ppm) against several Gram-positive and Gram-negative bacteria. The exact mechanism through which they present antimicrobial properties is yet to be elucidated [23].

Nanoparticles, due to their size, shape and surface chemistry are prone to interception by different defence components following entry into the body. For macrophage avoidance of nanoparticles several strategies that suppress the opsonisation processes can be undertaken. Thus, nanoparticle surface modification or coating with natural occurring complement inhibitors yield stealth or macrophage-avoiding nanoparticles [18].

Immunoglobulin G (IgG) is composed of four polypeptidic chains, two heavy and two light chains, linked through a disulphidic bond to give a ‘Y-shaped’ conformation. IgG has one Fc domain, which should be preferred in the adsorption onto the nanoparticle surface and two identical Fab domains responsible for antigen binding [24].

Calcium folinate (CF) is the salt form of folinic acid, a known drug used to combat the adverse effects of tetrahydrofolate reductase inhibitors [25].

The aim of this study was to develop a novel, silver based, bio-nanocomposite for biological applications. IgG molecule was chosen for the passivation of the AgNPs in order to avoid macrophage recognition of the synthesized structures. To our knowledge, this is the first report of an IgG-folinate functionalized AgNP bionanostructure developed for biological applications.

## Results and discussions

**Fig. 1:**
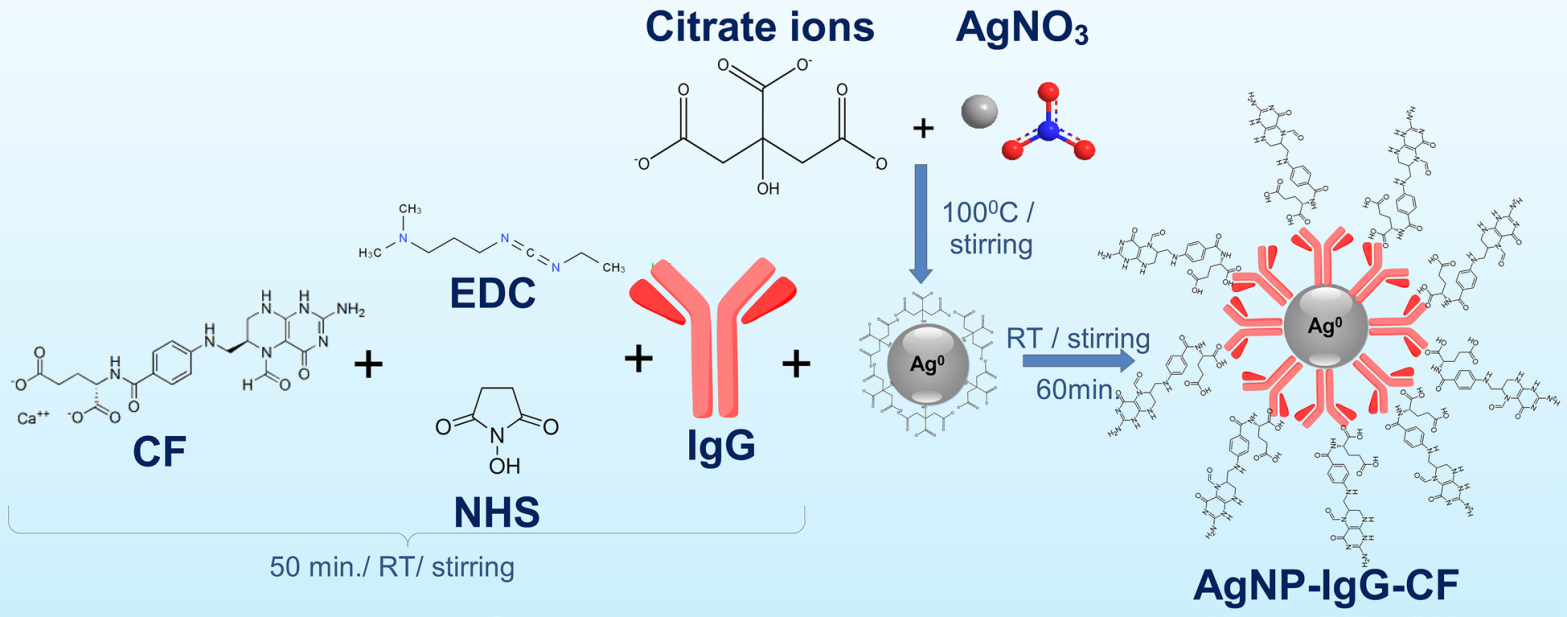
Graphical illustration of the synthesis protocol for the AgNP–IgG–CF bio-nanocomposite

The synthesis protocol used for obtaining the AgNP–IgG–CF bio-nanocomposite is presented in Fig. 1 and has three stages: in the first stage silver ions (Ag^+^) were reduced to metallic silver (Ag^0^) with the aid of sodium citrate; in the second stage IgG molecules were covalently coupled to the CF molecules and in the third stage silver nanoparticles were functionalized with the IgG–CF conjugate. The obtained functionalized nanoparticles in aqueous solution were kept at both room temperature (20 °C) and 4 °C for several weeks and did not present any agglomeration or sedimentation.

**Fig. 2:**
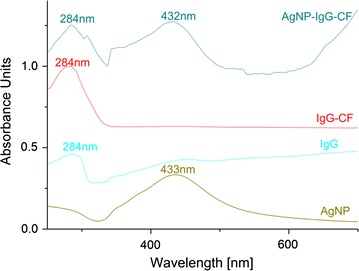
UV–Vis spectra for AgNP, IgG, IgG–CF and AgNP–IgG–CF samples

UV–Vis spectroscopy is a valuable technique in terms of size and morphology characterization of silver nanoparticle dispersions [26]. The UV–Vis spectra for the AgNP, IgG, IgG–CF and AgNP–IgG–CF samples are presented in Fig. 2. The citrate capped silver nanoparticles presented a surface plasmon resonance band (SPR) at 433 nm [27], while the IgG and the IgG–CF conjugate had a λ_max_ centered at 284 nm. In the case of AgNP–IgG–CF bio-nanocomposite the UV–Vis spectra two absorbtion peaks can be observed. The first peak, situated at 432 nm, is attributed to the SPR band of silver nanoparticles [28–30], while the second peak, at 284 nm, is attributed to the IgG molecules [31] coupled to the surface of AgNPs in the functionalization step. Available literature data shows that the SPR profile of AgNPs is dependent to the protein concentration and the surrounding media [20].

**Fig. 3:**
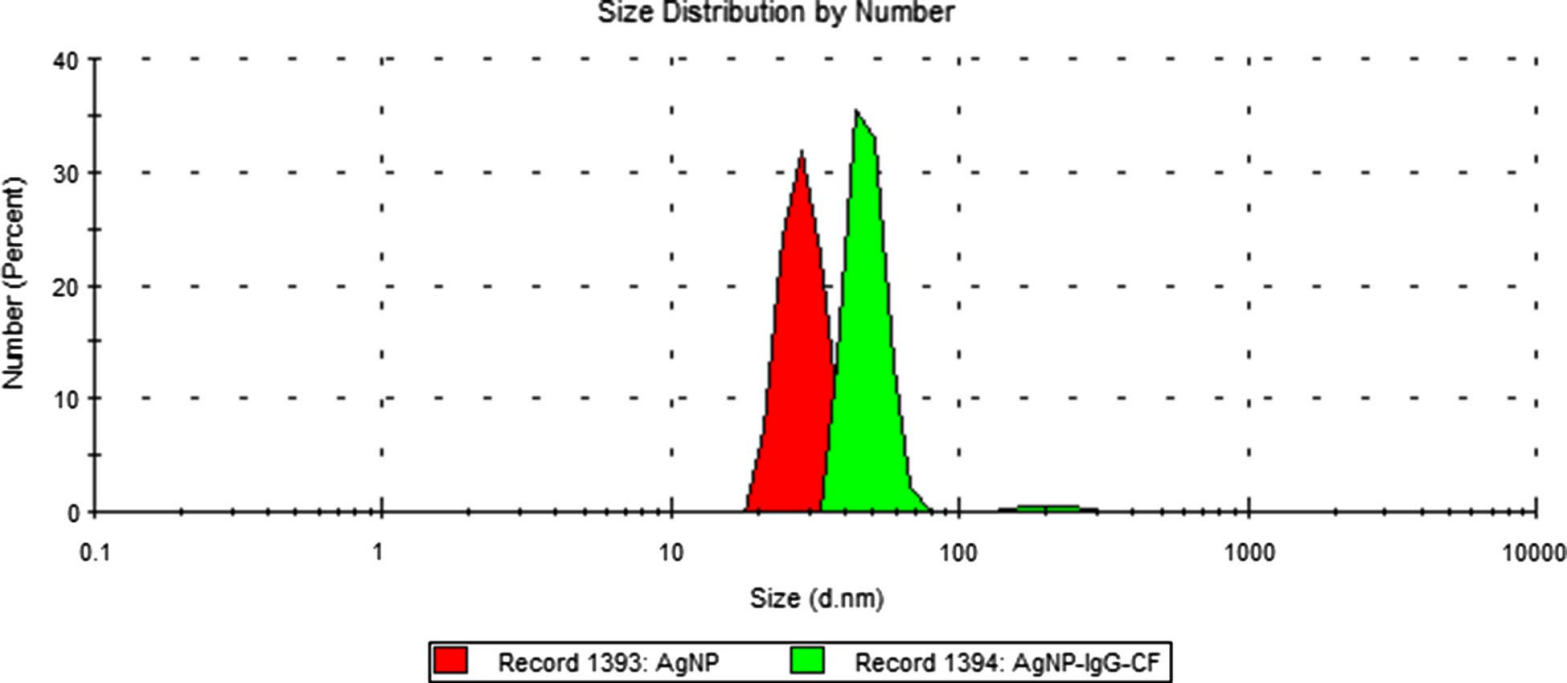
Dynamic light scattering size distribution curves for AgNP (*red*) and AgNP–IgG–CF (*green*) samples

Dynamic light scattering (DLS) measurements were performed in order to investigate the size of the citrate capped silver nanoparticles and IgG–CF functionalized AgNP. The DLS size distribution curves for the hydrodynamic diameters of AgNP and AgNP–IgG–CF samples are presented in Fig. 3. Both samples presented themselves as being monodisperse and stable at room temperature. For the AgNP a mean diameter of ~29 nm was registered, while the AgNP–IgG–CF had a mean hydrodynamic diameter of ~48 nm. The polydispersity index for the AgNP–IgG–CF sample was 0.283, indicating a narrow size range.

The typical dimensions reported in literature for IgG are approximately 14.5 nm × 8.5 nm × 4 nm [32], this corroborated with the DLS data, suggests that IgG has formed a monolayer on the surface of AgNPs during the functionalization step. Also the binding site of the immunoglobulin to the silver nanoparticle surface seems to be in the Fc domain, leaving the two Fab regions available for antigen binding.

**Fig. 4:**
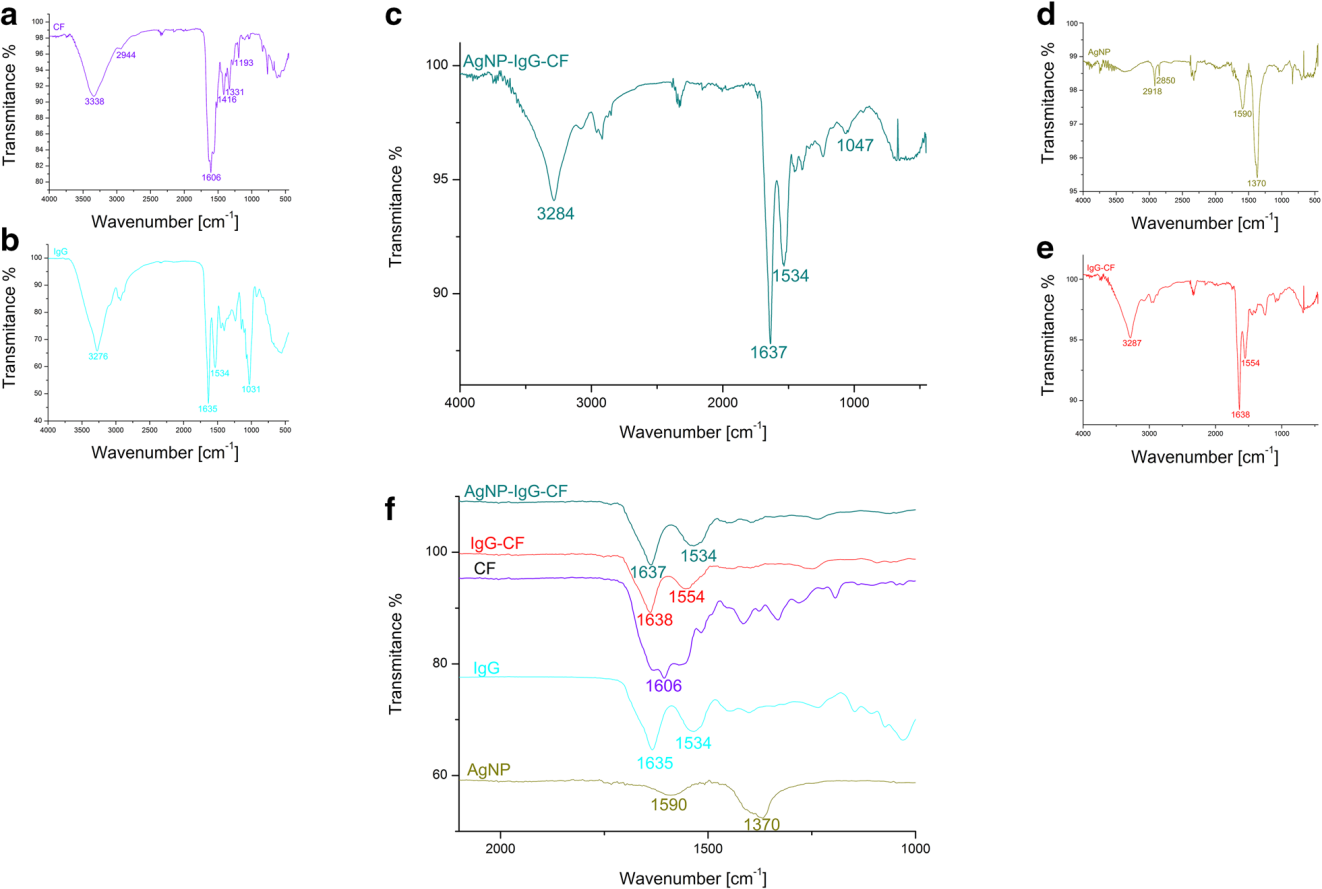
ATR-FT-IR spectra for: **a** CF; **b** IgG; **c** AgNP–IgG–CF; **d** AgNP; **e** IgG–CF samples in the region 4000–450 cm^−1^; and **f** comparison between the samples in the 2500–1000 cm^−1^ region

Attenuated total reflectance Fourier transform infra-red (ATR-FT-IR) spectroscopy is a straightforward technique that gives valuable information about the secondary structure of proteins [31]. Figure 4 depicts the IR spectra of (A) calcium folinate, (B) immunoglobulin G, (C) IgG–CF functionalized silver nanoparticles, (D) citrate capped silver nanoparticles and (E) IgG–CF conjugate. Figure 4f shows a comparison, in the 2500–1000 cm^−1^ region, between the IR spectra presented in Fig. 4a–e. In the case of the AgNP sample, the absorbtion bands at 1590 and 1370 cm^−1^ are attributed to the antisymmetric and, respectively symmetric stretching vibrations of COO^−^ from citrate molecule present on the surface of silver nanoparticles [33, 34]. For the IgG and IgG–CF samples, the IR bands at 1635–1638 and 1534–1554 cm^−1^ correspond to the amide I band (which leads to stretching vibrations of the C=O bond of the amide) and, respectively to the amide II band (C–N stretching and N–H bending vibrations) from the IgG β-sheets [31, 35]. These absorption bands are also present in the final bio-nanocomposite at 1637 and 1534 cm^−1^, making the IR spectra of the IgG and AgNP–IgG–CF nearly identical. Thus, in the case of the AgNP–IgG–CF sample, the disappearance of the IR band attributed to citrate ions, corroborated with the appearance of IgG characteristic IR bands confirm the successful functionalization of silver nanoparticles with the IgG–CF conjugate.

**Fig. 5:**
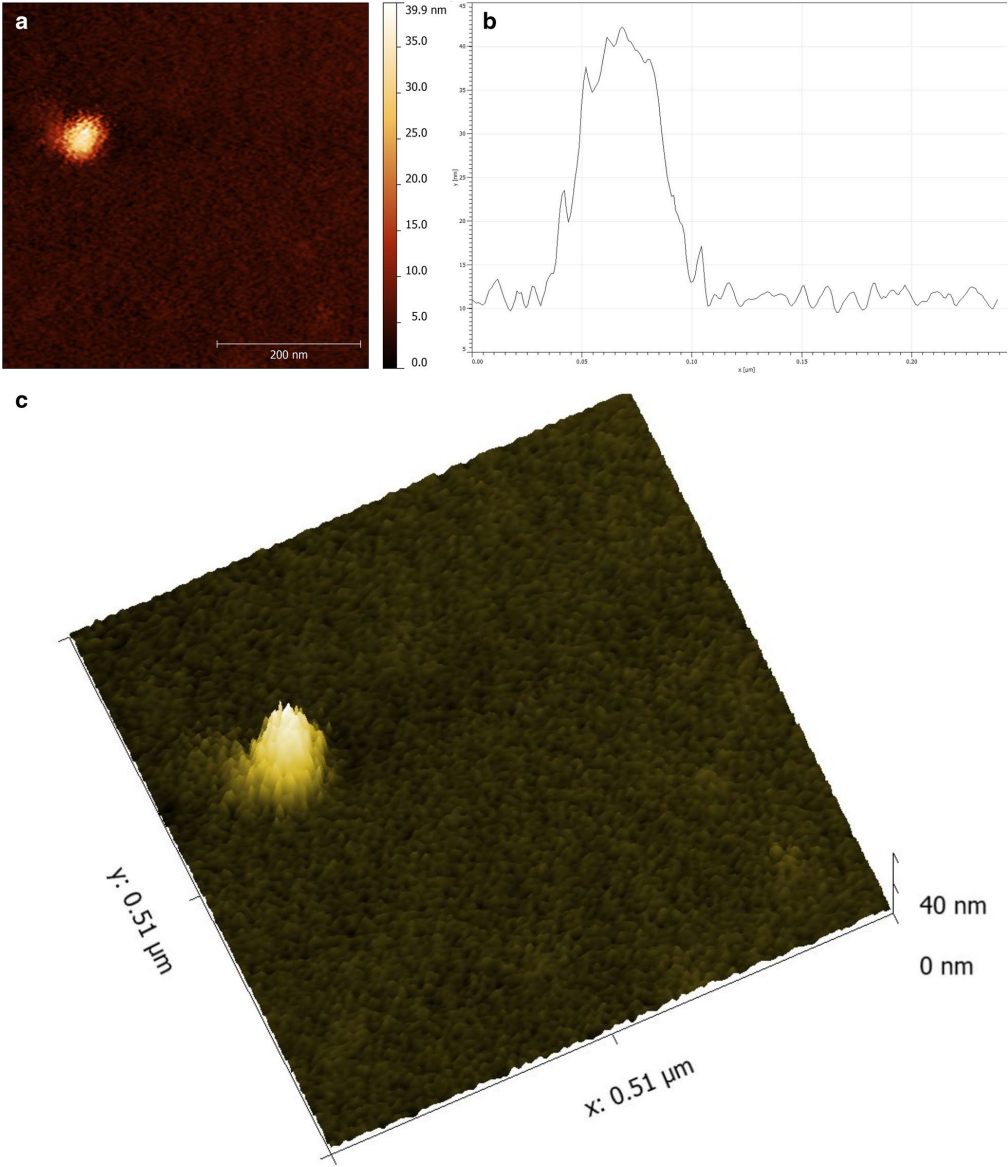
AFM measurement of AgNP–IgG–CF: **a** 2D image (*scale bar* 200 nm); **b** cross section graph of a single AgNP-IgG-CF nanoparticle; **c** 3D rendering of AgNP–IgG–CF

Atomic force microscopy was conducted in order to further investigate the size and shape of the AgNP–IgG–CF bio-nanocomposite. Figure 5a is a 2D representation of a single AgNP–IgG–CF nanoparticle, while Fig. 5c is a 3D representation of the same bio-nanostructure. AFM data collected showed that AgNPs were spherical in shape and had a mean diameter of ~26 nm. The IgG–CF functionalized silver nanoparticles were ~39 nm in diameter. The difference between the DLS and AFM data regarding nanoparticle size can be attributed to the fact that the dynamic light scattering technique provides a hydrodynamic diameter of the nanoparticle and the associated solvation layers [36].

## Experimental

Silver nitrate (AgNO_3_ 99.9 %), sodium citrate (≥99 %), *N*-(3-Dimethylaminopropyl)-*N*′-ethylcarbodiimide hydrochloride (EDC) and *N*-Hydroxysuccinimide (NHS) were purchased from Sigma-Aldrich™ (Darmstadt, Germany) and were used without further purification. Immunoglobulin G (IgG) was purchased from Kedrion (Lucca, Italy) and was purified with the aid of Pierce^®^ centrifugal concentrators with a 30 kDa molecular weight cut-off. Calcium folinate (CF) was purchased from Actavis (Romania). All glassware used was cleaned with aqua regia (HCl:HNO_3_, 3:1) prior to use.

Aqueous stable silver nanoparticles (AgNPs) were obtained by reducing silver ions in the presence of sodium citrate. Thus, 9 mg AgNO_3_ were dissolved in 50 mL H_2_O dist. and the solution was heated to boiling point. Next, 2 mL of sodium citrate (0.5 %) were quickly added under vigorous stirring and the reaction was allowed to continue until the solution reached a pale-yellow colour.

In order to covalently bind the immunoglobulin G with the calcium folinate, 3 mL IgG solution (5 mg/mL) were reacted with 1.5 mL EDC solution (30 mg/mL) and 1.5 mL NHS sol. (30 mg/mL) under continuous stirring for 20 min, at room temperature. Afterwards, 1 mL CF (3 mg/mL) was added and the reaction allowed to continue for another 30 min. For the purification of the IgG–CF conjugate, centrifugal concentrators with a molecular cut-off of 30 kDa were used at 4000 RPM for 60 min. The purified IgG–CF was re-eluted in 6 mL H_2_O dist. and 4 mL AgNP sol. were added under vigorous stirring, the reaction was allowed to continue at room temperature for 60 min. Finally, the obtained AgNP–IgG–CF bio-nanocomposite was separated by means of centrifugation at 13,200 RPM for 20 min and re-dispersion in H_2_O dist.

UV–Vis spectroscopy measurements were recorded with a Shimadzu UV-1800™ instrument. UV–Vis spectra were recorded for the AgNP, IgG, IgG–CF and AgNP–IgG–CF samples in the 800–200 nm range, with a spectral resolution of 0.5 nm. The OriginLab^®^ 7.0 software was used for normalization of the registered spectra.

Dynamic light scattering (DLS) data was registered on a Zetasizer—Nano S90 instrument (Malvern Instruments, Westborough, UK) at 20 °C and a 90° diffraction angle; a refractive index of 0.20 and a material absorption of 3.980 were considered.

Universal attenuated total reflectance Fourier transform infra-red spectroscopy (UATR-FT-IR) measurements were performed on a Perkin-Elmer Spectrum Two^®^ instrument equipped with a diamond ATR stage. All registered spectra were processed with the aid of the Spectrum 10™ software.

Atomic force microscopy (AFM) was conducted of a Workshop TT-AFM^®^ instrument (AFMWorkshop, CA, USA), equipped with ACTA-SS cantilevers (AppNano, CA, USA) operated in vibrating mode. The samples were deposited on a mica substrate with the aid of a KLM^®^ SCC spin-coater. The registered data was processed with the aid of the Gwyddion^®^ 2.36 software.

## Conclusions

Monodisperse IgG-folinate functionalized silver nanoparticles with sizes around 39 nm were obtained. UV–Vis and UATR-FT-IR spectroscopies were employed to confirm the successful functionalization of the silver nanoparticles. AFM and DLS measurements gave information about the size and shape of the nanoparticles prior and after the passivation with IgG. Immunoglobulin G formed a monolayer around the nanoparticles with the binding site seemingly in the Fc domain, leaving the two Fab regions available for antigen binding. To our knowledge, this is the first report of an IgG-folinate functionalized AgNP bionanostructure developed for biological applications. Further investigations are being carried out regarding the AgNP–IgG–CF interactions in biological systems.

## Abbreviations

AgNP: silver nanoparticles
CF: calcium folinate
IgG: immunoglobulin G
SPR: surface plasmon resonance
ATR-FT-IR spectroscopy: attenuated total reflectance Fourier transform infra-red spectroscopy
DLS: dynamic light scattering
AFM: atomic force microscopy
